# Specific Visualization of Glioma Cells in Living Low-Grade Tumor Tissue

**DOI:** 10.1371/journal.pone.0011323

**Published:** 2010-06-30

**Authors:** Sven R. Kantelhardt, Wouter Caarls, Anthony H. B. de Vries, Guy M. Hagen, Thomas M. Jovin, Walter Schulz-Schaeffer, Veit Rohde, Alf Giese, Donna J. Arndt-Jovin

**Affiliations:** 1 Department of Neurosurgery, Georg-August-University of Göttingen, Göttingen, Germany; 2 Laboratory of Cellular Dynamics, Max Planck Institute for Biophysical Chemistry, Göttingen, Germany; 3 Department of Neuropathology, Georg-August-University of Göttingen, Göttingen, Germany; Kings College London, United Kingdom

## Abstract

**Background:**

The current therapy of malignant gliomas is based on surgical resection, radio-chemotherapy and chemotherapy. Recent retrospective case-series have highlighted the significance of the extent of resection as a prognostic factor predicting the course of the disease. Complete resection in low-grade gliomas that show no MRI-enhanced images are especially difficult. The aim in this study was to develop a robust, specific, new fluorescent probe for glioma cells that is easy to apply to live tumor biopsies and could identify tumor cells from normal brain cells at all levels of magnification.

**Methodology/Principal Findings:**

In this investigation we employed brightly fluorescent, photostable quantum dots (QDs) to specifically target epidermal growth factor receptor (EGFR) that is upregulated in many gliomas. Living glioma and normal cells or tissue biopsies were incubated with QDs coupled to EGF and/or monoclonal antibodies against EGFR for 30 minutes, washed and imaged. The data include results from cell-culture, animal model and *ex vivo* human tumor biopsies of both low-grade and high-grade gliomas and show high probe specificity. Tumor cells could be visualized from the macroscopic to single cell level with contrast ratios as high as 1000: 1 compared to normal brain tissue.

**Conclusions/Significance:**

The ability of the targeted probes to clearly distinguish tumor cells in low-grade tumor biopsies, where no enhanced MRI image was obtained, demonstrates the great potential of the method. We propose that future application of specifically targeted fluorescent particles during surgery could allow intraoperative guidance for the removal of residual tumor cells from the resection cavity and thus increase patient survival.

## Introduction

About 77% of primary malignant central nervous system (CNS) tumors are classified as gliomas. In the USA about 18,000 cases of glioma are diagnosed every year and about 13,000 patients die of this disease annually[1]. Following the definition of the world health organization (WHO) gliomas are classified by their aggressiveness in grades from I to IV [2]. The more aggressive grades (III and IV) are also termed high-grade gliomas, whereas grade II tumors are termed low-grade. The pilocytic astrocytoma of the young adult and children is the only glioma WHO grade I (benign). Despite advances in surgical procedures and adjuvant therapies, the prognosis of malignant brain tumors remains poor. Gross surgical resection of high-grade gliomas has been demonstrated in prospective controlled trials to extend the survival of glioma patients significantly [3], [4], [5] (evidence level I). No level I evidence exists for low-grade gliomas. However, recent studies (retrospective case-series, evidence level V) favor early surgery and support a radical removal of diffuse low-grade gliomas if achievable at an adequate risk level [3], [4]. Most recurrent high- and low-grade gliomas arise from the primary site of the glioma or within the directly adjacent brain tissue. The longer survival time after more complete resection as well as the frequent recurrence in the area of the primary site suggest that the recurrent gliomas arise from remaining primary tumor cells in or close to the wall of the resection cavity.

Application during surgery of 5-aminolevulinic acid (5-ALA), which is metabolized to fluorescent protoporphyrin IX, was shown to increase ”total resections” from 36% to 65% as defined by loss of post-operative MRI contrast-enhancing tissue [6]. However, doubts persist as to the efficacy of the identification and resection of microscopical tumor remnants in the penumbra of the dye, in as much as the fluorescent agent is not restricted to the tumor cells but is found in the intracellular space where it can freely diffuse [7]. Furthermore 5-ALA does not stain low-grade gliomas, which are even more difficult to discriminate from the surrounding brain tissue because of the smaller change in cell morphology and the lack of MRI contrast.

Genome-wide profiling of archival glioma samples have revealed that Her1 (epidermal growth factor receptor, EGFR) expression and/or gene dosage is upregulated in >40% of all gliomas and ∼90% of WHO grade IV glioblastoma multiforme tumors (GBM)s [8], [9], [10], [11] as well as low grade oligodendroglial tumors [12] compared to normal adult brain. Cell biological experiments have shown that EGFR can be specifically labeled on live cells with fluorescent nanoparticles, semiconductor quantum dots (QD) [13], [14]. In this report we demonstrate that QDs specifically targeted to EGFR, can clearly distinguish low-grade as well as high-grade glioma tissue from normal brain tissue both at the macroscopic and the single cell level with very high contrast ratios in ex vivo experiments. The strong, photostable fluorescence and rapid differential binding of these probes meet some of the criteria required by surgeons to distinguish tumor cells left in the resection cavity.

## Results

### Kinetics of binding of QD-EGF in cultured glioma cells and alternative staining with MAbs and QD-GAMIG

**Figure 1:**
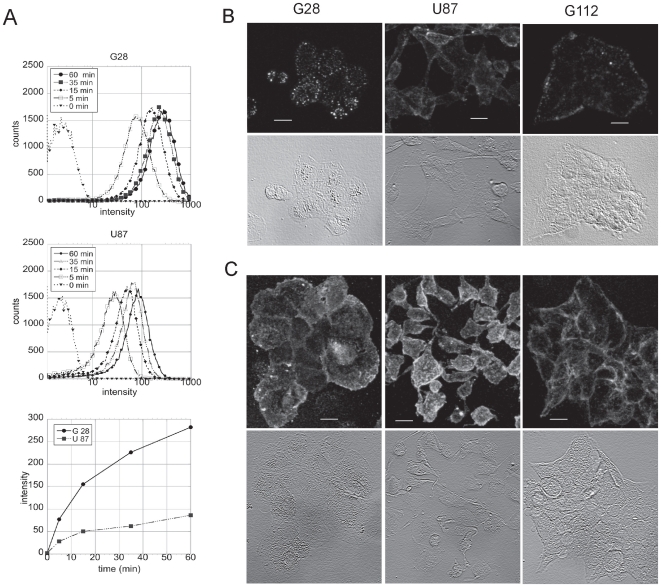
Specific targeting of EGFR on glioma cell lines by QDs. (A) Flow cytometric analysis of uptake of 655QD-EGF by cell line G28 (upper panel), and U87 (middle panel) at 37°C. Histograms for the time points 0, 5, 15, 35 and 60 min are displayed on a log scale. Excitation, 488 nm, emission 635–675 nm. Lower panel, mean intensity values for the various time points. Non-targeted QD staining of the cells gave the same fluorescence values as the unstained cells and are plotted as the 0 time point. Confocal fluorescence and phase images of monolayer cells after staining with (B) 2 nM 655QD-EGF or (C) MAb H199.12 antibody and 625QD-goat anti-mouse F(ab)_2_. Zeiss 510 Meta CLSM imaging. Excitation 488 nm, emission 615–700 nm and 595–680, B and C respectively, with a 40X water immersion, NA 1.2 objective. Fluorescence images of QD-EGF (A) on G28 and U87 cells lines were acquired with the same sensitivity and G-112 with a 10-fold higher laser intensity, whereas images in (C) were acquired at 595–680 nm with only a 4-fold difference in sensitivity for G-112. The images are maximum intensity projections of 0.5 µm optical sections after background substraction and median filtering. Bar, 20 µm.

QD-EGF (mono-biotinylated EGF coupled to Streptavidin-(PEG)- QDs, Invitrogen) was applied to cultured human glioma cell-lines in monolayer culture at 37°C. QDs lacking conjugated EGF were not taken up by any of the cell lines. The kinetics and extent of uptake was quantitated for G-28 and U87 cell lines by flow cytometry (Fig. 1A). Extensive uptake was achieved in less than 30 minutes. Staining of three-dimensional spheroids derived from G-28 and U87 cells maintained in non-adherent tissue culture conditions resulted in penetration and uptake of QD-EGF at least 2 or 3 cell layers into the spheres (data not shown). In a survey of 15 established human glioma tissue cell lines we observed that binding of QD-EGF varied considerably, consistent with the known variability of wildtype and mutant EGFR expression in gliomas. The EGFR mutant vIII lacks the EGF binding site but is constituitively active and is expressed in ∼60% of high-grade gliomas EGFR [15]. Such tumors will not bind QD-EGF but should be targeted by antibodies against the ectordomains of EGFR. We tested lines expressing EGFR with three different monoclonal antibodies (MAb) directed against various epitopes in the ectodomain of the EGFR (528, H-11, and 199.12) and QD-coupled secondary antibodies. A comparison of staining for the lines G-28, U87 and G-112 using the two procedures is shown in Figure 1B,C. Note that the image of the G-112 line stained with QD-EGF was acquired at 10x the laser power as the images for lines G-28 or U87 (Fig. 1B) whereas all imaging conditions were identical for QD-MAb staining (Fig. 1C). Fixed cell lines were also tested by normal immunofluorescence staining for EGFR expression with antibodies directed against either the extracellular or the cytoplasmic domain of the receptor as well as by cell lysis and western blot analysis (see Supplementary Figure S1). QDs without EGF or anti-EGFR Mab coupling did not bind to the cell lines nor did QDs coupled with isotypic but unrelated antibodies such as to Her2 or CMV.

### QD-EGF and QD-MAb staining in the orthotopic glioma model

**Figure 2:**
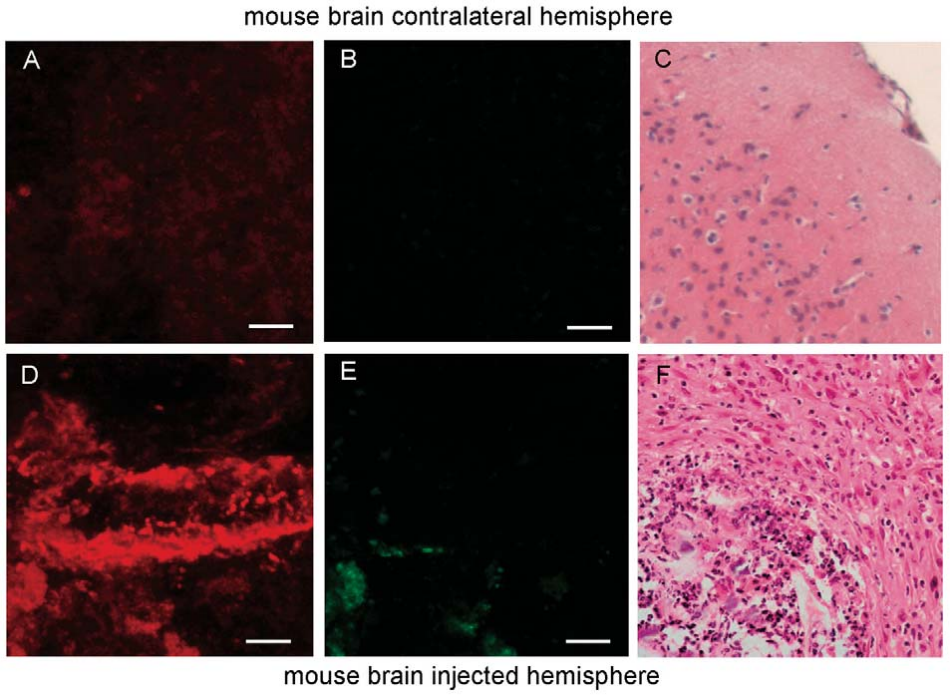
QD-EGF staining of an orthotopic mouse brain tumor. (A,B) Tissue sections from control contralateral hemisphere of a mouse injected with G28 human glioma cells and stained with 625QD-EGF. (D,E) Tumor tissue from the injected hemisphere. (A,D) Maximum intensity projections of 25 confocal optical sections at 1 µm intervals of the intensities 595-659 nm; (B.E), 530/20 nm (autofluorescence), Zeiss Meta CLSM, excitation 488 nm, 20×0.5 NA objective. Tissue imaged in panels A and B were treated identically to that in panels D and E and thereby constitute controls for the specificity of staining. (C,F) H & E staining and sectioning of tissue from the same hemispheres for pathology. Data were acquired with the same sensitivity and are not contrast stretched; some pixels in image D are saturated. Bar is 20 µm.

G-28 and U87 experimental glioma bearing mouse brains were explanted and coronary sections were stained immediately at 37°C. White light illumination of the explanted mouse brains showed a distorted anatomy, a loss of normal white and gray matter structures of normal mouse brain, and increased tissue volume at the site of the tumor implantation. Samples from tumor tissue and from the nonimplanted contralateral hemisphere were stained with QD-EGF for the G-28 tumors and monoclonal antibody coupled QDs (QD-MAb H199.12) for the U87 implanted mice, respectively. Tissue samples were examined by confocal microscopy. As in the cell cultures, G-28 cell line derived tumors in mouse brain showed extensive uptake of QD-EGF (Fig. 2A,D) and no uptake of untargeted QDs. Three-dimensional data stacks demonstrated a penetration of QD-EGF to about 20-30 µm into the tumor tissue slices. Tissue from the uninjected brain hemispheres stained with the targeted QD probes did not bind these or untargeted probes as shown in Figure 2B,E. After fluorescence imaging, all samples were forwarded for histo-pathological examination. Conventional H&E (haematoxylin and eosin) staining of sections from the QD imaged tissue (not necessarily corresponding to the same areas imaged) demonstrated the presence of solid and highly cellular tumor tissue in the samples positive for specific QD-fluorescence, whereas no tumor was detected within the contralateral hemisphere (Fig. 2C,F). Similar results were obtained with the U87 cell-line derived gliomas using QD-MAb199.12 (Supplementary Movie S1).

### QD-EGF and QD-MAb staining of human glioma biopsies

**Figure 3:**
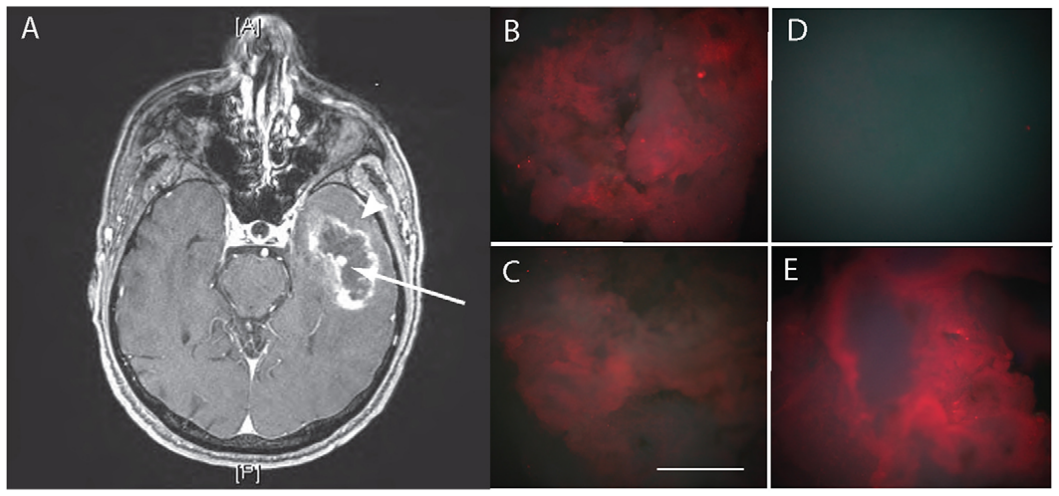
MRI and QD-probe digital macroimages from glioblastoma multiforme, grade IV biopsy X. (A) T1 weighted MRI axial scan showing gadolinium positive signal. (B–E) Digital macrophotographic images of ex vivo stained biopsies from the resected tumor and adjacent brain tissue stained with targeted QD probes taken with the same magnification and the same exposure times. Tumor tissue (B) 625QDStAv-biotin-MAb528 EGFR staining, (C) 625QDGAMIG-MAb 199.12 EGFR staining. Adjacent brain, (D) 625QDStAv-biotin-MAb528 EGFR staining, (E) invading tumor tissue, 625QDStAv-biotin-MAb528 EGFR staining. Excitation, 365 nm; emission >450 mm. Objective 5X NA 0.15. Bar 1 mm. Note that panel D serves as the control for panel B, ie stained with the same probes under identical conditions.

Human brain tumor biopsies were taken during standard neurosurgical resection of two glioblastoma multiforme WHO grade IV (GBM), one anaplastic astrocytoma WHO grade III and one oligodendroglioma WHO grade II. The high-grade tumors were located such that presumably unaffected, brain tissue had to be explanted during the surgical access (partial temporal lobectomy), providing control brain tissue for the staining under identical conditions with targeted QD probes as the tumor tissue. Both tumor tissue and normal brain tissue were also stained with control QDs lacking the EGF or primary antibody conjugation or conjugated to non-expressed epitopes and showed no binding. In the cases of the low-grade tumors where no normal brain tissue was resected, the tumor tissue was stained with non-targeted, streptavidin-(PEG)-QDs and goat-anti-mouse IgG coupled QDs for control. The biopsy sites of one of the GBM cases are illustrated on the preoperative gadolinium enhanced MRI scans in Figure 3A and for the second GBM see Supplementary Figure S2A (the arrow indicates the MRI positive tumor mass and the arrowhead, the adjacent brain tissue). The tissue specimens were placed on ice for a short period to transport the samples from the operating room to the laboratory before staining at 37°C. Samples of tumor tissue and brain were incubated with QD-EGF, as well as MAb QD-528, QD-H11, and QD-H199.12 at 37°C. In the case of both GBMs, areas of solid tumor and relatively unaffected adjacent brain tissue were easily distinguished macroscopically by visual observation after staining or with a normal digital photographic camera under UV Hg-halide light illumination (Fig. 3B,C (tumor) compared to D (adjacent brain); Suppl. Fig. S2B compared to C). H &E staining of the respective tissues (e.g. Suppl Fig. S2D and E) confirmed the identities of the tumor and normal tissues. Staining the biopsied (macroscopically) supposedly tumor-free tissues at the edge of the resection cavity with QD-MAb528 EGFR revealed the presence of remaining tumor cells not detected at the resolution of the enhanced MRI image in one of these tissue specimens as shown in Figure 3E (compare to 3D) and confirmed by post processing H&E staining.

**Figure 4:**
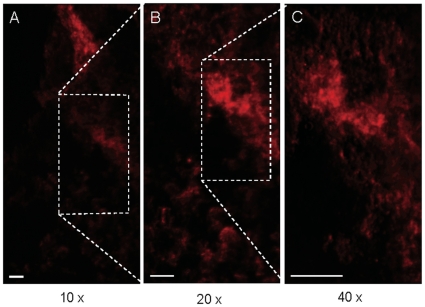
Three magnifications of a tissue specimen from human GBM Y stained with QD-MAb-EGFR. Maximum intensity projections of PAM acquired conjugate (confocal) images stained with 655QDGAMIG-MAb 199.12 EGFR. (A) 36 1-µm optical sections, 10X NA 0.3 objective; (B) 26 1- µm optical sections, 20X NA 0.5 objective; (C) 18 1- µm optical sections, 40 X NA 0.75 objective. Excitation 488 nm, emission 655/40 nm. Boxes denote the area enlarged in the next corresponding image. Bar 20 µm. Control for the staining shown in this figure is seen in Supplementary Fig. S2, panel C.

**Figure 5:**
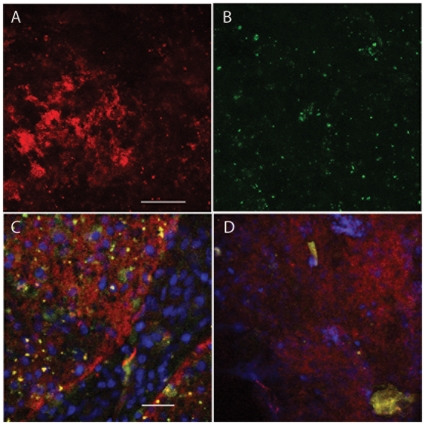
Staining of GBM X with EGFR targted QDs. (A,B) Maximum intensity projection of 19 2-µm confocal sections through the tumor tissue stained with 625QDStAv-biotin-MAb528 EGFR, excitation 488. A QD emission 595–649, B autofluorescence emission 520–553. Field in panels A & B, 230 µm, bar 50 µm. Controls for these probes are shown in Fig. 3, panel D. C tumor tissue stained with 625QDGAMIG-MAb 199.12 EGFR, counterstained with DRAQ5 for DNA. Excitation 488, QD emission 595–649, red; autofluorescence 520–552, green. DRAQ5 excitation 633, emission 660–745 nm, blue. D tumor tissue stained with 625QDStAv-biotin-EGF, counterstained with DRAQ5. Excitation and emission wavelengths and size as in C. Field in panels C & D, 153 µm, bar 25 µm. Objective 20X NA 0.5.

**Figure 6:**
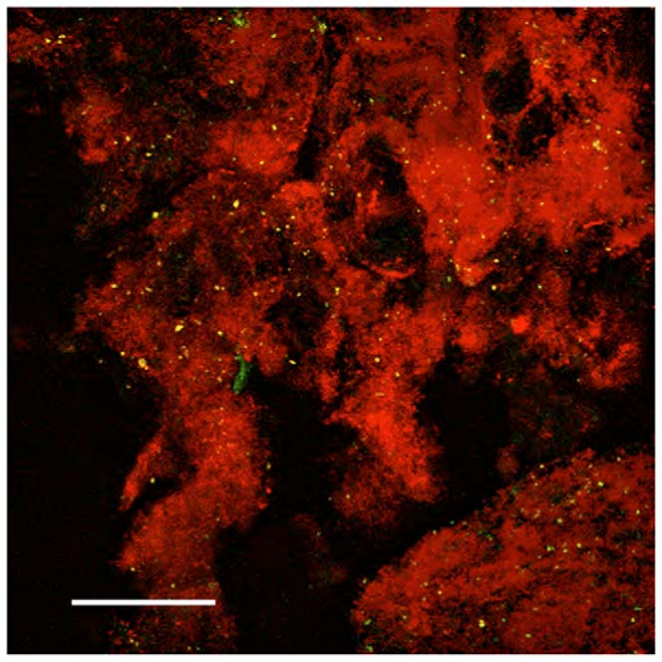
Staining of invading tumor cells in GBM X with EGFR targted QDs. Maximum intensity projection of 40 confocal 2-µm optical sections through tissue adjacent to tumor area with presumed invading tumor tissue (tissue as shown in Figure 3E) stained with 625QDStAv-biotin-MAb528 EGFR, excitation 488. QD emission 595-649, autofluorescence emission 520-553. Objective 20X, NA 0.5. Field 460 µm, Z depth 80 µm, bar 100 µm.

Both GBM QD-stained biopsies were imaged in a confocal scanning Zeiss 510 Meta microscope and a high-speed Programmable Array optical sectioning (PAM) microscope at higher resolutions. The latter is a versatile wide-field fluorescence microscope using patterned illumination and detection that achieves high-speed, single-molecule sensitive imaging [16], [17], [18]. Specific uptake of QD-EGF in individual tumor cells could be discerned at increasing magnifications (10X, 20X, and 40X) as shown for a single tissue specimen (Fig. 4). In the case of GBM X some of the tumor tissue was necrotic, showing distorted nuclei and no uptake of QD-EGF although it was positively stained by QD-MAbEGFR (Figs. 5 and 6). PAM images of QD-EGF probed tissues were used to quantitate the very high specific QD-fluorescence in the tumor compared with the adjacent brain samples which showed no specific uptake (see for example Suppl. Fig. S5A, tumor, compared to S5C, adjacent brain). The fluorescence intensities in the QD emission (635–675 nm) channel were >10^3^ times higher for the tumor tissue than those recorded from the normal brain tissue. Similar results were obtained by staining with the three QD-MAbs with contrast ratios between 200 and 1000 between tumor and brain biopsy tissues using the same probes on the two different tissues. Extensive imaging was performed on tissue stained with QD-EGF, QD-MAb-528 and QD-MAb-H199.12, Figs. 3,4,5,6; Supplementary Movies S2, S3, S4; and Supplementary Figs. S3, S4, S5 for both tumors. Counterstaining with DAPI or DRAQ5 demonstrated that the tumor tissue had a much higher density of nuclei than the tissue from the non-gadolinium positive region of both GBMs (e. g. Suppl. Fig. S5B,D).

All samples were processed for conventional histo-pathological examination after completion of the fluorescence imaging studies. Histology confirmed elements of a glioblastoma WHO grade IV in specimens that showed specific QD fluorescence, whereas the tumor-adjacent brain samples contained predominantly grey and white matter (Suppl. Fig. S2C and E). Results from both high-grade GBMs were similar and supported the hypothesis that QDs targeted to EGFR can specifically recognize glioma cells *ex vivo*.

**Figure 7:**
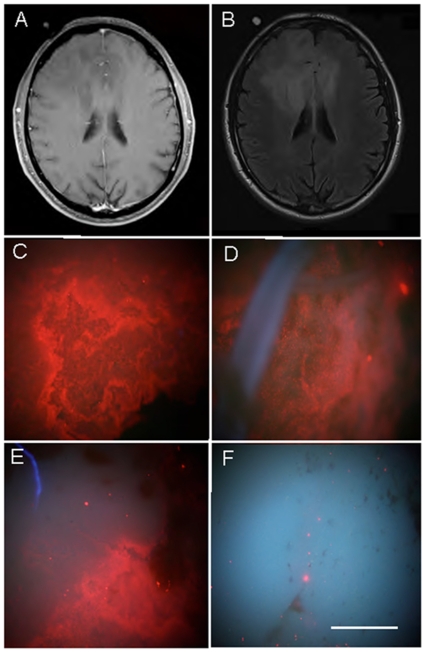
MRI and QD-probe digital macroimages from an astrocytoma III. (A) T1 weighted MRI axial scan with no gadolinium positive signal. (B) FLAIR MRI of the same section as in a. (C–F) Digital macrophotographic images of ex vivo stained biopsies from the resected tumor stained with targeted (C–E) QD probes or untargeted QDs taken with the same magnification and the same exposure times. (C) 625QDStAv-biotin-MAb528 EGFR staining. (D) 625QDStAv-biotin-EGF staining. (E) tumor margin 625QDStAv-biotin-MAb528 staining. (F) uncoupled 625QDStAv staining. Excitation, 365 nm; emission >450 mm. Objective 5X NA 0.15. Bar 1 mm.

**Figure 8:**
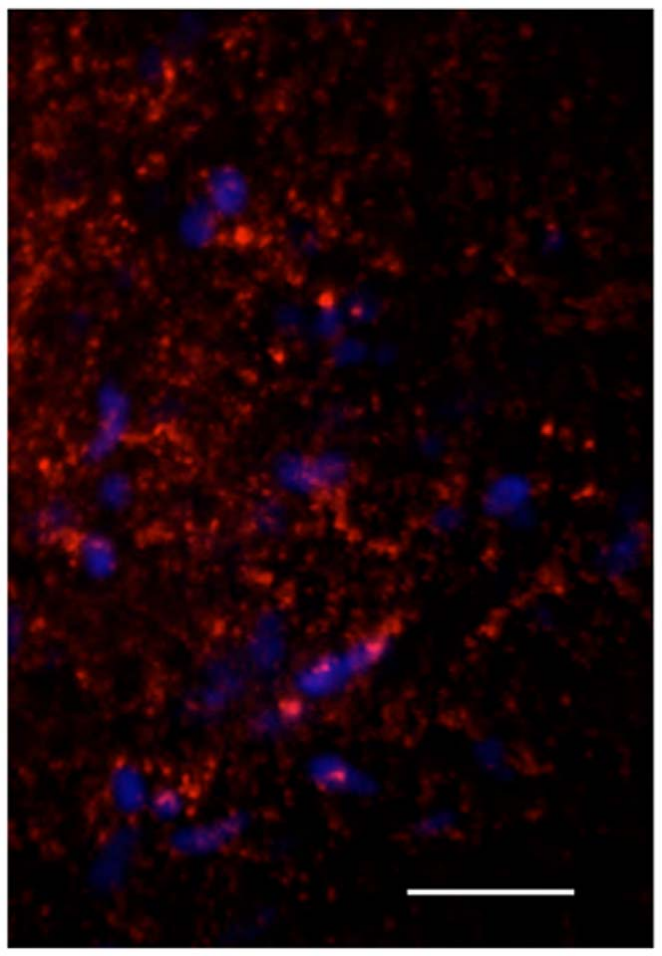
QD-EGFR targeted staining of astrocytoma biopsy. PAM sectioned maximum projection image of 11 slices at 2 µm intervals. 625QD-biotin-MAb528EGFR excitation 488 nm, emission 625/40 nm and DRAQ5 excitation 658 nm, emission 700/40 nm overlay. Bar, 25 µm. Control staining is shown in Fig. 7, panel E.

More important to the surgeon is whether targeted QDs can delineate low-grade gliomas where no discrete boundaries are visible in MRI scans and no uptake of 5-ALA occurs. Therefore, we applied similar probes as those described above to grade II and grade III biopsy tissue. The first sample described was an anaplastic astrocytoma WHO grade II/III which showed no MRI galodinium contrast enhancement but only evidence of inflammation (Fig. 7A T1 and 7B FLAIR). The macro pictures of the stained tissue sections are seen in panels C-F. A control from the surrounding brain tissue could not be obtained in this case, because the superficial situation of the tumor did not require resection of adjacent brain. Tumor tissue was used as control and stained with non-targeted QDs (Fig. 7F). By microscopic examination of QD-EGFR targeted tumor, stained cells could be discerned scattered throughout autofluorescent tissue more closely resembling normal brain tissue (Fig. 7C). The high nuclear density and QD-MAb EGFR staining are shown in a reconstruction of 11 optical sections taken on the PAM (22 µm depth) in Figure 8, and another sample on the CLSM of a 24 µm depth reconstruction, Supplementary Movie S5. The neuropathological examination revealed scattered mitoses as signs of malignant transformation from WHO grade II to III throughout the biopsy. (5-ALA was not applied in this surgery).

**Figure 9:**
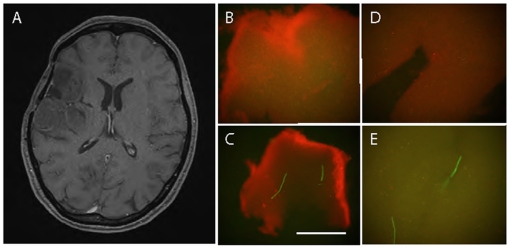
MRI and QD-probe digital macroimages from an oligodendroma stage II. (A) T1 weighted MRI axial scan with no gadolinium positive signal. (B–E) Digital macrophotographic images of ex vivo stained biopsies from the resected tumor stained with targeted (B–D) QD probes or untargeted QDs taken with the same magnification and the same exposure times. (B,C) 655QDStAv-biotin-MAb528 staining. (D) 655QDStAv-biotin- PDGFRα staining. (E) uncoupled 655QDStAv staining. Excitation, 365 nm; emission >510 mm. Objective 5x, NA 0.15. Bar 1 mm.

**Figure 10:**
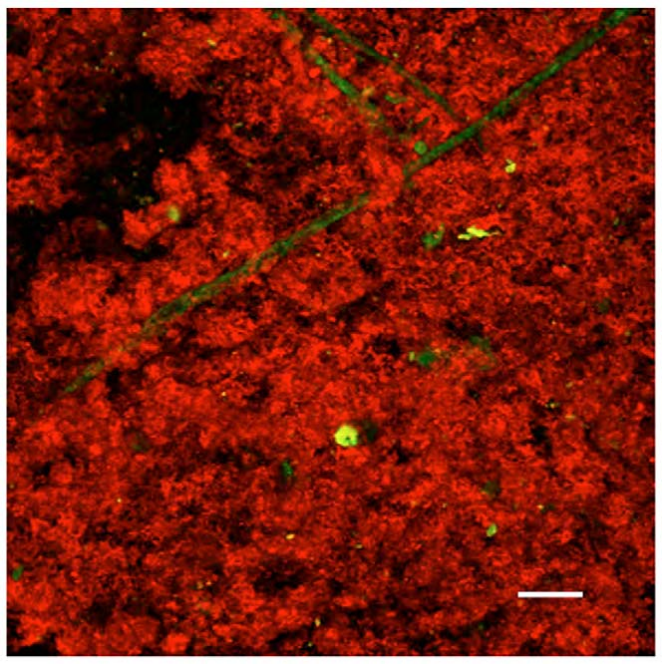
QD-MAb EGFR staining of an oligodendroglioma biopsy. Image of a Z projection, opacity 15%, ramp 40%,of 50 5-µm sections of the oligodendroglioma biopsy shown in Fig. 9C. 655QDStAv-biotin-MAb528EGFR, excitation 488 nm; emission >650, red, autofluorescence emission 530/20, green. Objective 10X NA 0.3. Field 921 µm, bar 100 µm.

A further case examined by QD-bioconjugate targeted staining was a low-grade glioma with no MRI contrast enhancement (oligodendroglioma WHO grade II) Figure 9A. Again no surrounding brain tissue could be resected during surgery. However staining with QD-MAb-EGFR resulted in a 200-fold elevated tissue fluorescence (Fig. 9B,C), quantitated at higher magnification (see Fig. 10), compared to the autofluorescence or staining with uncoupled QD as control (Fig. 9E). The tumor tissue was also weakly positive for targeted QD-MAb-PDGFα (Fig. 9D, Suppl. Fig. S6); PDGFα being a cell surface marker that has been linked to oligodendroglioma tissue [12], [19], [20]. Strong staining by QD-MAb528 EGFR was seen throughout the entire 250 µm thickness of the small biopsy tissue (Fig. 9C) Figure 10, Supplementary Movie S6. A high nuclear density was seen in all of the QD positive areas as shown in Supplementary Figure S7A. Although some of the biopsy tissue could be stained throughout (Fig. 10) in many cases as stated previously the QD targeted probes can only penetrate about 3 cell layers due to the density of the tumor tissue and the size of the QDs. This is shown by a 3-D volume projection reconstruction (Suppl. Fig S7B) of the fluorescent image planes in which one can see QD-Mab-EGFR fluorescence extending to 26 µm (3 cell layers) and nuclei visible to 36 µm (an additional 1–2 cell layers). Individual tumor cells are clearly distinguishable at this magnification as seen by nuclei demarcated by surrounding QD-probe staining.

These data show conclusively that glioma tumor cells even in low-grade tumor biopsies expressing EGFR can be visualized at both macroscopic and microscopic magnifications by specifically staining with monoclonal antibodies against EGFR and/or EGF, and in some cases to monoclonal antibodies against PDGFR, coupled to quantum dots.

## Discussion

### QD and EGFR

QDs have unique advantages for cellular imaging [21]: (i) high absorption cross-sections and quantum yields, permitting detection down to the single nanoparticle level and reliable quantitative detection of binding and transport phenomena; (ii) extreme photostability, allowing imaging over prolonged periods; (iii) broad excitation spectra rising toward the UV, allowing the simultaneous excitation of different QDs; and (iv) narrow, tunable emission bands throughout the visible spectrum. QDs with the proper bioconjugation are not taken up non-specifically by cells but can be easily coupled to biomolecules targeting specific receptors, as some of us have demonstrated using living cultured cells [13], [22], [23].

The obvious marker for our QD probe was the epidermal growth factor receptor (EGFR or Her1), upregulated in many head and neck tumors and an established target for glioma therapy. The erbB tyrosine kinase receptor family (HER1-4) is important in the embryogenesis and development of the central nervous system. With the completion of the growth processes of the brain in adulthood the EGFR is down regulated. However, EGFR is involved in the tumorgenesis of gliomas [9], [24], [25], [26], [27].

The data presented here show highly specific labeling of native human glioma biopsies that can be distinguished from normal brain tissue down to the single cell level by staining with QD-EGF and/or QD-MAb anti-EGFR. The ability of the QD-conjugate to identify low-grade glioma biopsies constitutes to our knowledge the first specific *ex vivo* staining of low grade-glioma cells in tissue. The delineation of the tumor cells was apparent at all microscopy magnifications.

A survey of 15 human glioma cell lines showed that MAbs against the ectodomain of EGFR could positively identify lines that were negative for EGF binding due to mutations in the EGF binding site or lines with low receptor density. The cell line G-112 is one such line that bound Mab against EGFR but very weakly bound QD-EGF Fig. 1B,C and Suppl. Fig S1. In view of the upregulated Her1 (epidermal growth factor receptor) expression and/or gene dosage in >40% of all gliomas [9], [24], [25], [26], [28], [29], [30] as well as in low grade oligodendroglial tumors [12], we postulate that a cocktail of QDs attached to several MAbs against EGFR as well as QD-EGF would unambiguously distinguish these gliomas from normal human brain. Other possible cell membrane epitopes that are upregulated on glioma cells compared to normal brain tissue are PDGFRα [20], [30]and several integrins [31] which could also be considered for an even more general cocktail. In particular, low grade gliomas express a variety of glial progenitor markers concomitant with PDGFRα [20], [30].

Although the uptake of QDs and retention in animals has not led to adverse effects over periods of months [32], [33], there remain concerns about the toxicity of the core (CdSe) of semi-conductor QDs should the particles breakdown intracellularly. We expect that less- or non-toxic yet equally fluorescent nanoparticles based on Si [34], noble metals or dendrimers with long wavelength emission but benign chemical compositions will become commercially available. Chemical coupling of biomolecules to QDs and such particles can be effected without using streptavidin-biotin conjugates such that no antigenicity would be provoked by their use.

The PAM high-speed sectioning microscope is presently a research laboratory prototype. Newer designs that could be directly applicable to the surgical theater and provide single cell sensitivity are under development within an EU-funded project (http:www.mpibpc.mpg.de/fluoromag) and are expected to be ready for commercialization in the near future.

### Future prospects

This study has been restricted to glioma cell lines, mouse orthotropic tumors and ex vivo human biopsy material. The results demonstrate highly specific staining of tumor tissue compared with normal brain both in high-grade and low-grade biopsies. Presently there are no specific markers available to surgeons for directing resection of low-grade tumors. We anticipate in the future that targeted fluorescent nanoparticles will find use in directing resection guidance for low-grade glioma tumors similarly as 5′ALA staining is used in high-grade glioma resections presently.

While this paper was under review Nyugen et al. published a study in which fluorescent particles with sites for MMP2 enzyme cleavage were used for labeling orthotopic mammary tumors in mice for improved surgical resection[35]. While MMP2 is frequently upregulated in tumors it is also present in normal tissue and the differential staining achieved in this study was a maximum of 13.6-fold.

The use of highly fluorescent-nanoparticle conjugates for identification of brain tumor cells could be extended to targeting molecules for specific histotyping of brain tumors, as demonstrated for the PDGFα-receptor in the oligodendroglioma sample. It may even be possible to analyze the functional states of tumor tissue with such reagents. Stem-cell markers have identified a subpopulation of glioma cells that show aggressive tumor growth associated with a poor prognosis [20], [36]. Identification of specifically aggressive clusters of cells would allow their selective removal. In summary, we foresee the emergence of new, smart nanoparticles for gene expression-dependent resection guidance.

## Materials and Methods

### Cell culture and orthotopic glioma mouse model

The human glioblastoma derived cell lines G-112, G-28 [37], [38] and U87 (source, ATCC) were grown in MEM containing 10% FCS. All cell lines were free from mycoplasma. For intracranial implantation in nude NMRI mice, cells were harvested from monolayer culture by trypsin digestion. Cells were washed and resuspended at a concentration of 2×10^4^/µl. Prior to the implantations, animals were anaesthetized by peritoneal injection of ketamine/xylazine solution (200 mg ketamine and 20 mg xylazine in 17 ml of saline) at 0.15 mg/10 g of body weight. For the procedure the cranium was fixed in a stereotactic frame (TSE Systems, Bad Homburg, Germany). A 1 mm burr hole was placed 3 mm lateral to the bregma and a stereotactic implantation of 3 µl cell suspension injected over 3 min was placed in an area corresponding to the internal capsule 0.5 mm below the tracts of the corpus callosum. Following implantation, 50 mg/kg novaminsulfone was administered s.c. and 1 mg/ml novaminsulfone was added to the drinking water for three days to relieve postoperative pain. Four weeks post implantation, tumor-bearing brains were explanted following a lethal intraperitoneal injection of 50 mg/kg xylazine and 350 mg/kg ketamine. Coronal sections of the mouse brains were performed immediately and the sections were processed for EGFR-QD staining. Animal studies were approved by the animal study referee of the Georg-August-University in Göttingen and the animal research commission of Germany in Braunschweig.

### Human tumor biopsies and histologies

The ethical committee of the Georg-Ausgust University, Göttingen, gave permission for staining of glioma tissue removed during standard neurosurgical procedures in conjuction with our optical tissue analysis project. Informed written consent was given by all patients included in this study. The human biopsies were taken during standard neurosurgical procedures. Biopsy sites were in the central tumor mass and, where possible, in tumor adjacent brain as defined by image guided neuronavigation. They were registered by neuronavigation and correlated to the preoperative MRI-scans (3T, Magnetom Trio, Siemens Medical Solutions, Erlangen, Germany). Human brain tumor biopsies were immediately processed for EGFR targeted QD-staining. Following the imaging studies all specimens were formalin fixed and the tissue blocks were sectioned parallel to the optical imaging plane. The tissue blocks were paraffin embedded and 5 µm sections were cut. Standard H&E staining was performed. All samples were graded by a neuropathologist.

### QD coupling with EGF and monoclonal antibodies

Mouse monoclonal antibodies (MAb) 528 (IgG_2a_), H-11 (IgG_1_) and H199.12 (IgG_2a_), specific for the extracellular portion of human EGFR (Her1), were obtained from Dianova or purified from monoclonal cell culture supernatant by Protein G Sepharose chromatography. H-11 and H199.12 recognize both wt EGFR and EGFRvIII. Mouse monoclonal antibody CD140a (biotinylated anti- PDGFRα (IgG_1_) was purchased from BioLegend. QD-EGF ligand was formed by incubation of biotin-EGF (Molecular Probes) with 20 nM streptavidin conjugated, pegylated 655QDStAv (Q10121MP), 625QDStAv (A10196), or 705QDStAv (Q10161MP) (Invitrogen) at a 3∶1 ratio at 4°C with mixing for >30 min in PBS with 0.5% BSA. MAbs were either directly conjugated to amino-QDs (Invitrogen) or QDStAv (for biotinylated 528 and PDGFRα) or staining was carried out in 2 steps using MAb followed by QD-coupled goat anti-mouse (Fab)_2_ (GAMIG) (Q11021MP or A10195) (invitrogen). The peak emission wavelength of the QDs is denoted throughout the text.

### Staining of cell cultures and tissue samples

Glioma cell lines were plated on glass coverslips or in coverglass chamber slides (Lab-Tek, Nunc) and stained in vivo for 30 min at 37°C by 2 nM QD-EGF or 5 µg/ml MAb against the extra-cellular portion of Her1 followed by either conventional fluorophore labeled GAMIG or QD-coupled GAMIG. Imaging was performed on the live cells or after fixation in 3.7% paraformaldehyde (results were the same for either condition). For kinetic studies on the rate and extent of QD-EGF uptake, cells were incubated for the times specified in the text, harvested by trypsin, fixed in paraformaldehyde and analyzed by flow cytometry. Both unlabeled cells and cells incubated with QDs without EGF gave the same peak shown as the zero time point.

Tissue slices obtained from animal models or surgical biopsies were stained *ex vivo* in Tyrode's buffer containing 1% BSA at 37°C containing 4 nm QD-EGF or QD-MAb for 30 min with gentle agitation followed by 3 changes of Tyrode's/BSA for 15 min or 30 min of 10 mg/ml MAb followed by washing and subsequent staining with 10 nM QD-GAMIG and washing. The scheme for ex vivo staining is shown Supplementary Fig. S8. Tissue slices were either imaged directly or fixed in 3.7% paraformaldehyde for 24 hrs and kept in PBS in 4-well Lab-Tek coverglass chamber slides for macro and microscopic imaging. (A preliminary report of the staining of a single GBM by QD-EGF was presented at the SPIE International BIOS conference, January 24, 2009 [39]).

### Macroscopic observation

Tissue sections were excited by epi-illumination with an X-Cite Hg-halide lamp (EXFO, Mississauga, Ontario) at 365 nm through a 10X NA 0.3 objective of an Olympus IX71 inverted fluorescence microscope and observed with a 410 nm or 510 nm longpass emission filter on a Canon EOS 40D camera attached to the camera port of the microscope. Photographs were also taken from above the sample, with a Canon EOS 40D camera with a zoom EF 70–200 mm f/4 L IS, EF 1.4x II extender and 500 D close-up lens.

### Confocal laser scanning microscopy

A Zeiss CLSM 510 Meta microscope was used for imaging immunofluorescent and QD labeled cell lines and tissue sections. Laser excitation wavelengths were 488 nm for QD fluorescence, 532 nm for Cy3 and 633 nm for Cy5. Cell monolayers were imaged with a 40X NA 1.2 water immersion objective. QD labeled tissue sections were imaged with a 10 X NA 0.3 dry objective. In tissue labeled with QD 625, DNA was stained after fixation with 5 µM DRAQ5 (Biostatus Ltd, Leicestershire, UK) and imaged with HeNe laser excitation at 633 nm, emission >6501 nm.

### PAM, high speed sectioning microscopy

A Programmable Array Microscope [18] (PAM) prototype widefield optical sectioning microscope was used for sensitive high speed imaging of QD-targeted tissue slices. The PAM uses a ferroelectric liquid-crystal-on-silicon (LCoS, Fourth Dimension Displays, Dumferline, Scotland) reflective array to create structured patterns for excitation and emission. Both conjugate (emission largely from the focal plane) and non-conjugate (largely out of focus emission) images were acquired by an iXon DV885 emCCD camera (Andor, Belfast, Ireland) on an Olympus IX71 inverted microscope equipped with Prior XY and piezoelectric Nano Z stages using 10X NA 0.3, 20X NA 0.5, or 40X NA 0.75 dry objectives. Sectioned images were created in real-time from the combined conjugate and non-conjugate images. Excitation was with an argon ion laser at 488 nm and emission collected with 624/40, 655/40 nm or 705/40 nm (Chroma, Rockingham, Vermont) bandpass emission filters. DNA-DAPI images were recorded without sectioning using 365 nm excitation from a Hg/halide Exfo lamp and 450/40 nm emission. DNA-DRAQ5 images were obtained by HeNe laser excitation at 658 nm with emission at 705/40 nm.

### Flow cytometry

The cells stained for determination of the kinetic uptake of 655 QD-EGF were measured in a Coulter Epics Flow Cytometer with 488 nm argon ion laser excitation, emission at 655/40 using logarithmic PMT settings. The histograms represent 50,000 cells per time point with all cells measured at the same gain settings.

## Supporting Information

Figure S1 Western blot for EGFR expression of glioma cell lines shown in Figure 1 compared to Hela cells. EGFR was quantitated with rabbit polyclonal antibody 1005 (Santa Cruz Biotech) and tubulin with Mab 21D3 (gift from Mary Osborne) after HRP-secondary antibody binding and ECL (Pierce). G28 expresses 53%, U87 expresses 60% as many receptors as Hela and G-112, 5% as many.
(0.38 MB TIF)

Figure S2 Human glioblastoma multiforme, grade IV biopsy, Y. (A) Biopsy sites are denoted on the preoperative gadolinium enhanced MRI scan. The arrow denotes biopsy region in tumor tissue, arrowhead in adjacent brain. Digital macrophotographic images of the 655QD-EGF stained (B) tumor tissue, (C) brain tissue adjacent to the tumor site. Excitation, 365 nm; emission >450 mm. Objective 5X NA 0.15. Bar 1 mm. (D,E) Sectioned and H&E stained images of the same respective tissue specimens after fixation and embedding for pathology. Bar, 4 mm.
(1.20 MB TIF)

Figure S3 PAM image of QD-anti-EGFR staining of GBM Y. Biopsy stained with MAb 199.12 and 655QD-GAMIG. Maximum intensity project of 27 1-µm optical sections of tissue from GBM shown in Fig. 3d, some pixels are saturated. PAM image, 20X 0.5 NA objective, excitation 488 nm, emission 655/40 nm. Bar, 25 µm.
(1.03 MB TIF)

Figure S4 Stereopair of GBM Y staining by QD-EGF. Maximum intensity projection of 11 5-µm confocal optical sections (XY, 921 µm; Z depth, 55 µm) of tumor tissue stained with 655QDStAv-biotin-EGF. Excitation 488 nm, emission >650. Objective 10X, NA 0.3. Bar, 100 µm.
(2.24 MB TIF)

Figure S5 PAM images of anti-EGFR stained GBM Y biopsy and adjacent human brain tissue specimens. Human biopsy of a GBM and adjacent brain tissue stained with 655QDGAMIG-MAb 528 EGFR. (A) PAM images from the galodinium positive area and (C) from adjacent brain tissue; (B,D) widefield DAPI staining of nuclei in the surface cells of the same areas as shown in A and C respectively. (A) Maximum intensity projection of 23 1-µm optical sections of the QD-targeted tumor specimen. (C) Maximum intensity projection of 35 one µm sections of QD-targeted adjacent brain tissue. PAM images, 20X 0.5 NA objective, excitation 488 nm, emission 655/40 nm. Bar, 20 µm.
(0.97 MB TIF)

Figure S6 QD-anti-PDGFR targeted oligodendroma tissue. Maximum intensity projection of PAM images from 50 sections at 2 µm intervals through oligodendroglioma tumor tissue stained with 655QDStAv-biotin-MAb PDGFR (tissue shown in Fig 9D). Excitation 488 nm; emission, red, QD655 signal (655/40 nm); yellow, autofluorescence signal (550/70 nm). Objective 20X NA 0.5, bar 25 µm.
(0.52 MB TIF)

Figure S7 High nuclear density in QD-MAb-EGFR stained oligodendroglioma biopsy. (A) Maximum intensity projection through 22 µm of the oligodendroglioma biopsy after QD-MAb-EGFR staining, fixation and counterstaining with DRAQ 5 for DNA, excitation 633, emission >650 blue; 655QDStAv-biotin-MAb528EGFR,excitation 488, emission >585 red. Objective 20X NA 0.5. Field 153 µm, bar 25 µm. (B) Volume rendering (Image J plugin Volume View) of another field of the same tissue showing penetration of the targeted QD-probe up to 3 cell layers. The intensities for the Draq5 staining were enhanced in the deeper layers by 25% to compensate for fluorescence loss due to scattering in order to make the nuclei visible in this reconstruction. Field 153 µm xy, total depth 36 µm, QD signal visible to a depth of 28 µm.
(1.47 MB TIF)

Figure S8 Experimental scheme. Schematic depicting resection locations and ex vivo targeted QD staining of glioma biopsy tissues as performed in this study (A and B).
(0.32 MB TIF)

Movie S1 Orthotopic U87 glioma tissue from mouse brain.
(0.69 MB MOV)

Movie S2 Confocal laser scanning 3-D reconstruction image of QD-MAb-EGFR stained human GBM Y tissue specimen.
(0.79 MB MOV)

Movie S3 Confocal laser scanning 3-D reconstruction image of QD-MAb-EGFR stained human brain tissue specimen.
(0.65 MB MOV)

Movie S4 Confocal laser scanning 3D reconstruction image of GBM X stained with QD-EGF.
(0.91 MB MOV)

Movie S5 Confocal laser scanning 3D reconstruction image of an astrocytoma III stained with QD-MAb-EGFR.
(0.66 MB MOV)

Movie S6 Confocal laser scanning 3D reconstruction image of an oligodendoglioma stained with QD-MAb-EGFR.
(1.40 MB MOV)
