# Supplementary figures and images for: Specific Visualization of Glioma Cells in Living Low-Grade Tumor Tissue
Source: PLoS One. 2010 Jun 30;5(6):e11323. doi: 10.1371/journal.pone.0011323 (PMC2894859; doi:10.1371/journal.pone.0011323)

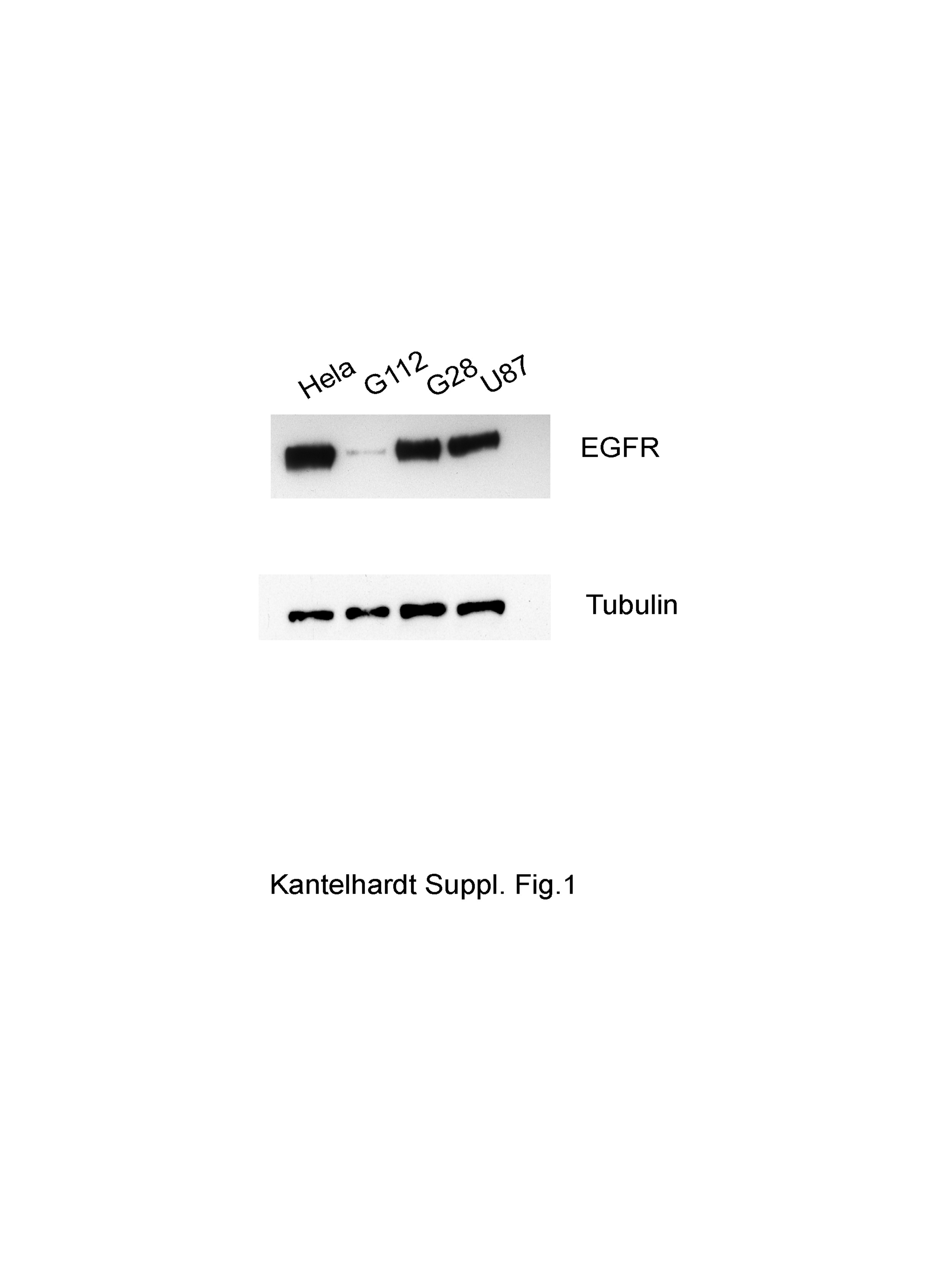

Supplement: Figure S1 — Western blot for EGFR expression of glioma cell lines shown in Figure 1 compared to Hela cells. EGFR was quantitated with rabbit polyclonal antibody 1005 (Santa Cruz Biotech) and tubulin with Mab 21D3 (gift from Mary Osborne) after HRP-secondary antibody binding and ECL (Pierce). G28 expresses 53%, U87 expresses 60% as many receptors as Hela and G-112, 5% as many. (0.38 MB TIF) [file pone.0011323.s001.tif]

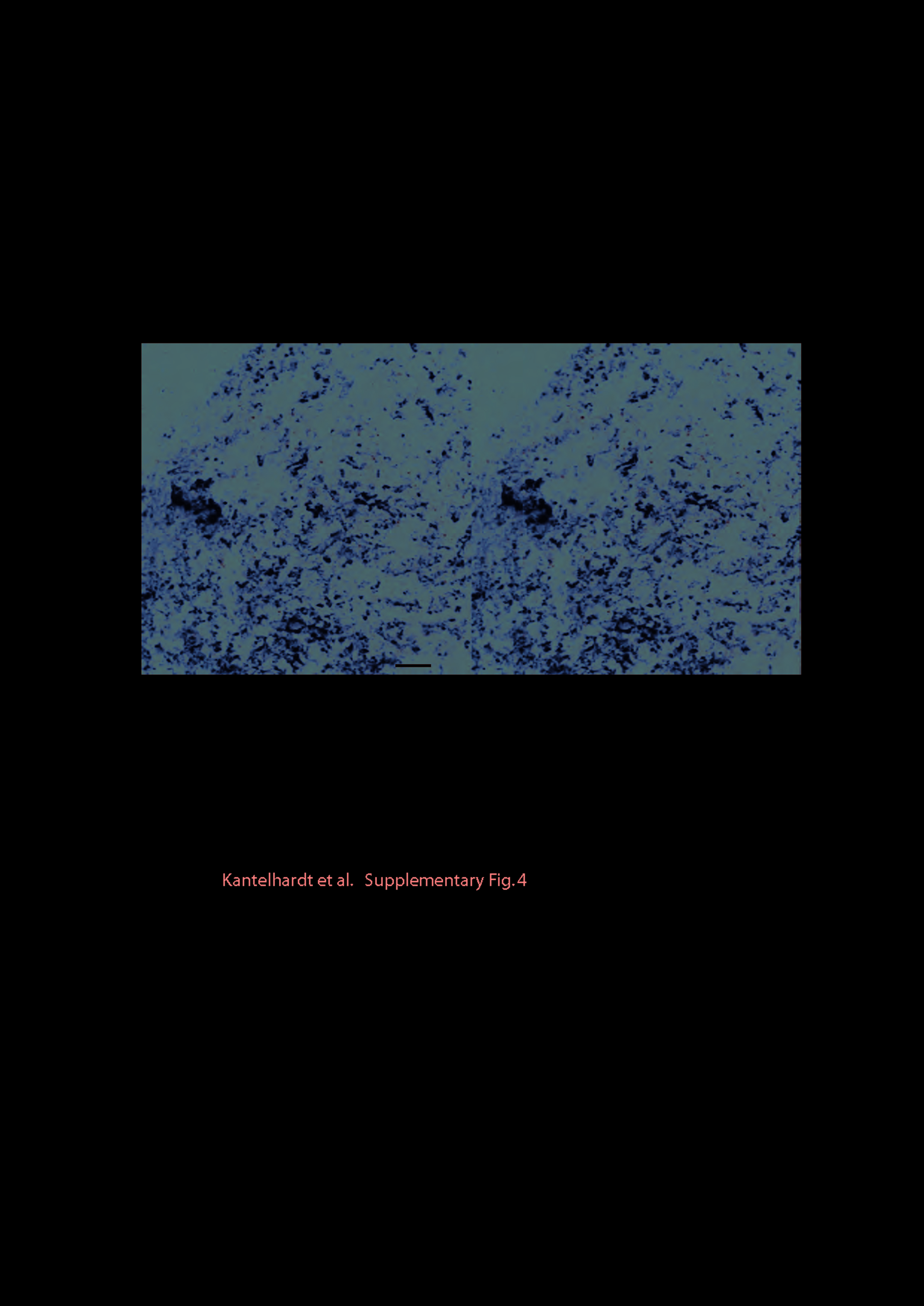

Supplement: Figure S4 — Stereopair of GBM Y staining by QD-EGF. Maximum intensity projection of 11 5-µm confocal optical sections (XY, 921 µm; Z depth, 55 µm) of tumor tissue stained with 655QDStAv-biotin-EGF. Excitation 488 nm, emission >650. Objective 10X, NA 0.3. Bar, 100 µm. (2.24 MB TIF) [file pone.0011323.s004.tif]

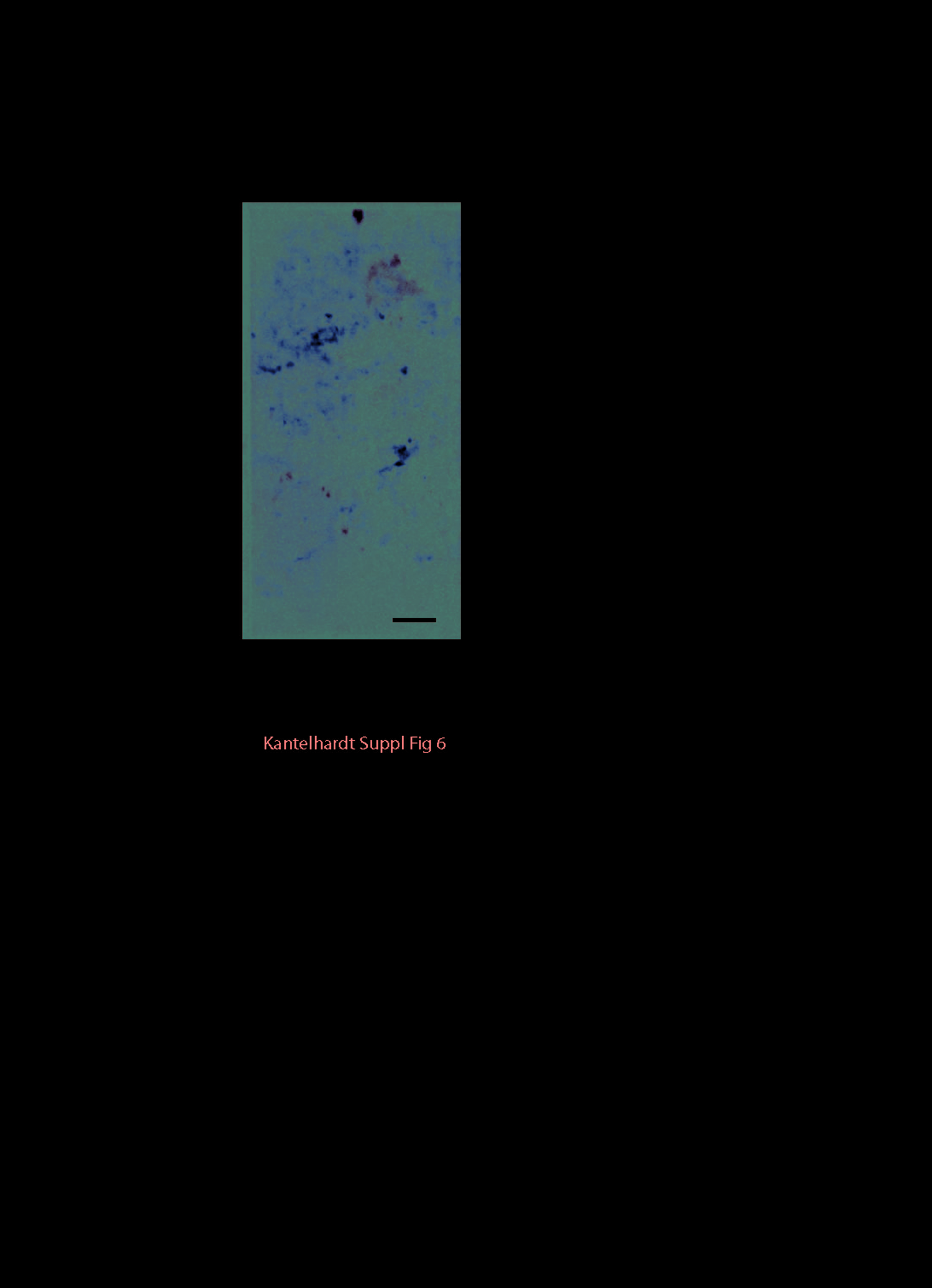

Supplement: Figure S6 — QD-anti-PDGFR targeted oligodendroma tissue. Maximum intensity projection of PAM images from 50 sections at 2 µm intervals through oligodendroglioma tumor tissue stained with 655QDStAv-biotin-MAb PDGFR (tissue shown in Fig 9D). Excitation 488 nm; emission, red, QD655 signal (655/40 nm); yellow, autofluorescence signal (550/70 nm). Objective 20X NA 0.5, bar 25 µm. (0.52 MB TIF) [file pone.0011323.s006.tif]

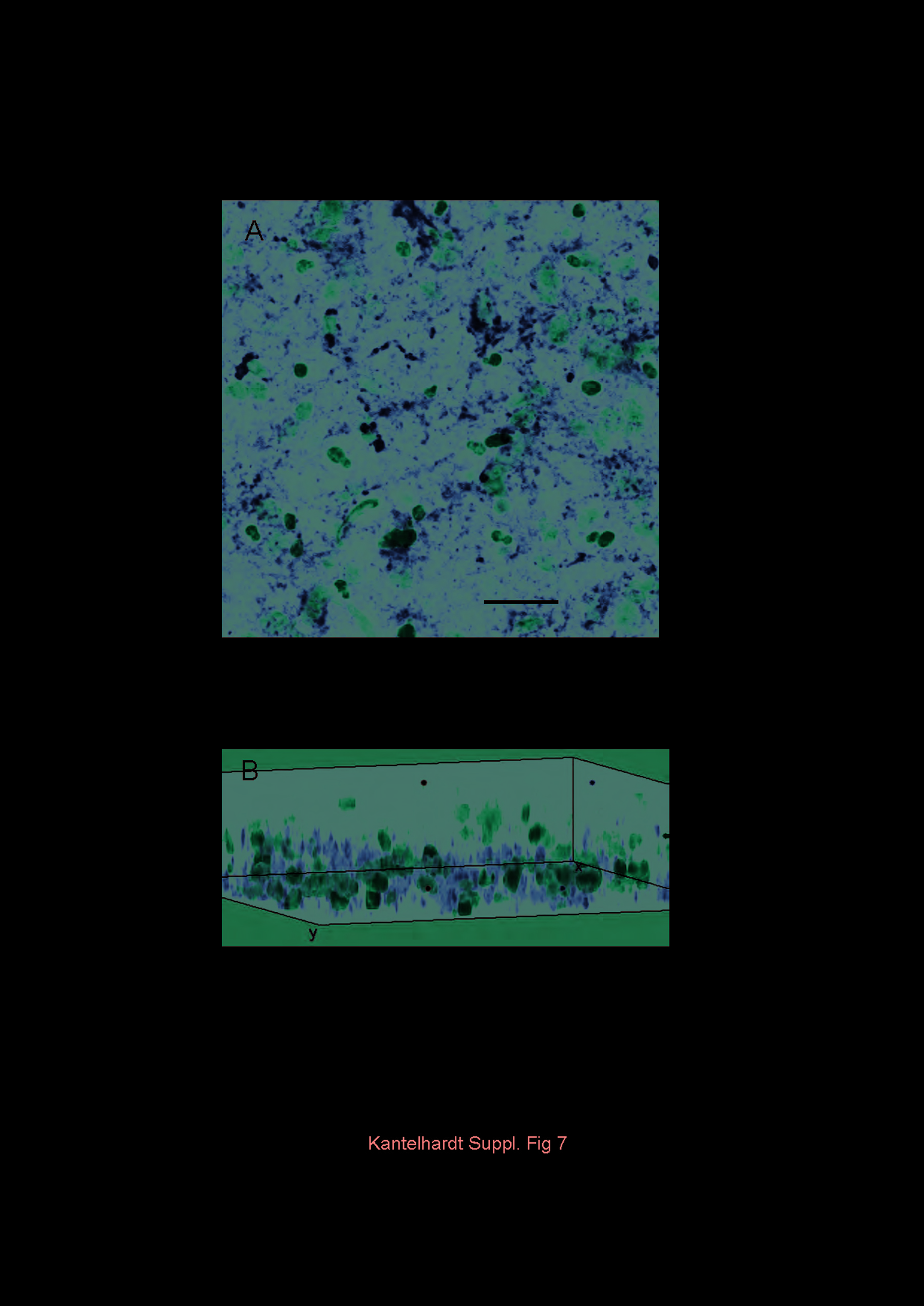

Supplement: Figure S7 — High nuclear density in QD-MAb-EGFR stained oligodendroglioma biopsy. (A) Maximum intensity projection through 22 µm of the oligodendroglioma biopsy after QD-MAb-EGFR staining, fixation and counterstaining with DRAQ 5 for DNA, excitation 633, emission >650 blue; 655QDStAv-biotin-MAb528EGFR,excitation 488, emission >585 red. Objective 20X NA 0.5. Field 153 µm, bar 25 µm. (B) Volume rendering (Image J plugin Volume View) of another field of the same tissue showing penetration of the targeted QD-probe up to 3 cell layers. The intensities for the Draq5 staining were enhanced in the deeper layers by 25% to compensate for fluorescence loss due to scattering in order to make the nuclei visible in this reconstruction. Field 153 µm xy, total depth 36 µm, QD signal visible to a depth of 28 µm. (1.47 MB TIF) [file pone.0011323.s007.tif]

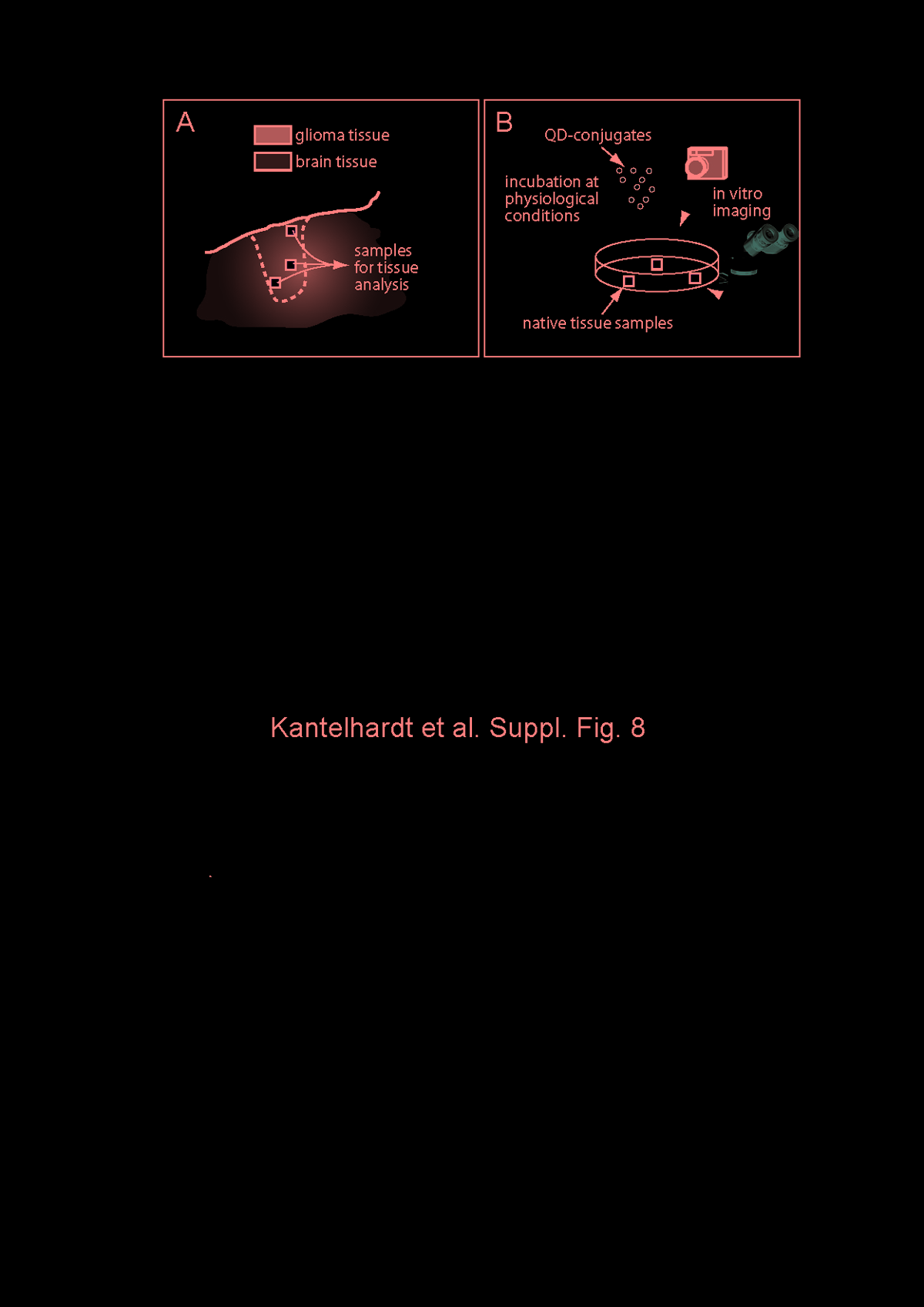

Supplement: Figure S8 — Experimental scheme. Schematic depicting resection locations and ex vivo targeted QD staining of glioma biopsy tissues as performed in this study (A and B). (0.32 MB TIF) [file pone.0011323.s008.tif]
